# Catering to patients with Parkinson’s disease: a survey on self-perceived competence and barriers of speech and language pathologists in Malaysia

**DOI:** 10.1136/bmjopen-2025-106250

**Published:** 2026-06-09

**Authors:** Giuditta Smith, Nursabrina Binti Kamarulzaman, Ching Sin Siau, Shin Ying Chu, Pei Jun Woo, Mike Minwen Zhang, Man-Ching Yuen, Maria Garraffa

**Affiliations:** 1 School of Health Sciences, University of East Anglia, Norwich, UK; 2 Universiti Kebangsaan Malaysia, Bangi, Malaysia; 3 Faculty of Health Sciences, Centre for Community Health Studies (ReaCH), Universiti Kebangsaan Malaysia, Bangi, Malaysia; 4 Department of Psychology, Sunway University, Bandar Sunway, Malaysia; 5 Department of Applied Data Science, Hong Kong Shue Yan University, Hong Kong, Hong Kong

**Keywords:** Parkinson-s disease, Speech pathology, Health Services for the Aged, Health Surveys

## Abstract

**Abstract:**

**Objective:**

Referrals to speech and language pathology are infrequent for people with Parkinson’s disease (PD), despite speech and communication being often affected and greatly impacting their quality of life. This study investigated the knowledge, self-competence and challenges faced by speech and language pathologists (SLPs) in Malaysia when managing PD cases.

**Design:**

Participants self-administered an online-survey in a cross-sectional study design. The survey consisted of 14 questions on current practices of SLPs with their patients with PD, self-perceived competence when assessing and managing PD and perceived barriers for catering to patients with PD. Inferential statistics were run on self-perceived competence across domains and their relationship with demographic/current practice factors. Descriptive statistics were used to analyse perceived barriers.

**Setting:**

The survey was administered in English through Google Forms.

**Participants:**

54 Malaysian SLPs with at least one active case of PD in their caseload were invited via email and WhatsApp Messenger. These contacts were obtained from the Speech-Language Therapists Association of Malaysia (SPEAK), and snowball sampling was encouraged to recruit additional SLPs through other social networks.

**Primary and secondary outcome measures:**

To quantify Malaysian SLPs’ self-perceived competence levels (assessed on 5-point Likert scales) in assessing and managing five key domains in patients with PD: speech, language, oro-motor skills, cognition and swallowing; and to identify the frequency and types of barriers encountered in clinical practice with patients with PD through structured multiple-choice questions. Secondary outcomes included quantifying current service delivery patterns (frequency of PD referrals, stage at referral, caseload size), multidisciplinary consultation patterns and confidence levels in managing rehabilitation risks associated with PD, all measured through structured survey items with categorical or ordinal response options.

**Results:**

Most participants had 1–5 patients with PD in their active caseload, referred at a middle or advanced stage of the disease. The majority of participants felt competent in assessing and managing motor speech and language in patients with PD. Conversely, most of them did not feel competent in assessing and managing cognition in these patients, regardless of demographic factors or current practices. This difference was significant. Most participants also reported facing barriers such as health conditions or comorbidities, family expectations on the therapy outcome and the unavailability of a multidisciplinary approach.

**Conclusion:**

The study reveals that SLPs working in Malaysia feel competent in working with motor speech and language in individuals with PD. However, it highlights a need for additional training to address cognitive assessment and management as a crucial tool to boost functional communication in people with PD. The study also reveals a need for promoting a multidisciplinary approach.

**STRENGTHS AND LIMITATIONS OF THE STUDY**

- Cross-sectional survey design employed validated questionnaire adapted from prior research and pilot-tested with ten subjects before implementation.
- Snowball sampling through the Speech-Language Therapists Association of Malaysia enabled recruitment of geographically diverse participants representing 14 states across all regions of Malaysia.
- Survey design combined quantitative Likert-scale assessments with multiple-choice questions to capture both competence levels and specific barriers.
- Self-reported data on competence and practices may introduce response bias and limit objective assessment of clinical capabilities.
- Small sample size of 54 participants limits generalisability to the broader Malaysian SLP population working with patients with Parkinson’s disease.

## Introduction

### Speech and language disorders in Parkinson’s Disease

 Global estimates show that over 8.5 million people were living with Parkinson’s Disease (PD) worldwide in 2019,^1^ with an age-standardised incidence rate of 13.43 per 100 000 and a growth of 159.73% since 1990.^2^ PD can be described as a loss of motor functions, with the most prominent symptoms including tremor, involuntary movement and painful muscle contractions. Several complications accompany PD, affecting both cognitive and speech and language skills in this population. Most notably, cognitive impairment affects almost a quarter of people with PD in the early-middle stages,^3 4^ often progressing to dementia in the late stages.^5 6^

Cognitive-communication skills encompass the mental processes essential for effective communication and interaction. These skills are fundamental to facilitating meaningful exchanges, incorporating key functions such as attention, memory, problem-solving and executive functions.^7^ Communication difficulties are prevalent in individuals with PD, affecting motor speech and language systems and contributing to social disengagement and heightened risks of isolation in these patients, ultimately reducing quality of life.^8^ As a consequence of the general loss of motor functions in patients with PD, one of the most common communication complications is motor speech disorder, with an estimated prevalence between 40% and 70%.^9^,^11^ Motor speech disorder in people with PD is mostly characterised by reduced vocal volume, imprecise articulation and monotony of pitch and loudness, affecting intelligibility.^12 13^ Communication difficulties not related to motor speech disorder are also reported in up to 90% of people with PD^14^ in the form of difficulties in lexical access,^15 16^ syntactic processing^17 18^ and in verbal fluency, information content and cohesion,^19 20^ greatly impacting their ability to effectively express their needs, wants and feelings and participate in society (i.e., their *functional communication* abilities). Functional communication is strongly correlated to cognitive status,^21^,^23^ and both are strong predictors of quality of life in people with PD,^23^,^29^ with most people experiencing feelings of frustration and inadequacy, often leading to withdrawal or avoidance from social interactions.^27 30^

### Speech and language pathology with PD

Speech and language pathologists (SLPs) are integral to the screening, assessment and treatment of people with PD. As such, SLPs working with people with PD should be prepared and confident in addressing motor speech impairments affecting intelligibility as well as cognitive and language disorders that frequently impact functional communication. However, assessment and intervention practices for PD are still mostly focused on motor speech only, and SLPs are still facing several barriers to service delivery in people with PD.^31 32^ First, not many patients with PD are referred to SLPs, despite showing impairments in speech and language: in a survey involving physical therapy clinics, occupational therapy clinics and speech and language pathology clinics in the Netherlands, for instance, only around 14% of patients with PD were in speech language therapy, as opposed to the over 60% who were in physical therapy.^31^ Around a third to half of people with PD have never accessed speech language therapy.^21 32^ Since the percentage of people with PD who show speech and language disorders is much higher, this shows that people with PD are being underserviced. Moreover, SLPs who see very few cases of PD in their caseloads have little hands-on experience. While multidisciplinary is perceived as crucial for managing people with PD and the lack of it is indicated as a real obstacle to equitable service provision, problems stemming from a lack of communication within the treatment team can result in less effective treatment delivery.^33 34^ For example, Swales *et al*^30^ highlighted that two-thirds of Australian SLPs did not have access to cognitive evaluations for individuals presenting with progressive neurological disorders, and this was a barrier to their management of cognitive-communication disorders effectively.

The multifaceted nature of the disorder is in itself challenging for comprehensive treatments. In a systematic review, Ciucci *et al*^35^ reveal several impediments to efficient treatment and diagnosis, from the health condition or comorbidities of the patients, to the lack of consistent training and guidelines in line with research suggestions. In the same review, some studies have pointed out the lack of an evidence base for their practices with PD individuals, particularly in cognitive-communication management, which may be due to most research being focused on motor speech disorder therapies, and less on cognitive-communication disorders.^34^ This is problematic for efficient treatment, given the tight connection between cognition and functional communication. Treatments on cognitive functions, particularly short-term memory, have already proven fruitful in boosting functional communication in aphasia, and their application to people with PD has been strongly recommended.^16 21 36 37^

### The context of Malaysia

At the time of writing, around 380 SLPs are actively working in Malaysia’s government and private sectors. Despite the long history of the speech language pathology profession in the country compared with other Southeast Asian countries,^37^ it still faces challenges due to a lack of supportive and informed multidisciplinary teams, infrastructure weaknesses,^36^ lack of training regarding evidence-based practices (EBPs) and enhancement of research skills in order to create and access EBPs,^38^ as well as a lack of assessment tools that are appropriate for the target language(s) of its multilingual population.^39 40^ While PD management is one of the mandatory courses for SLP certification, it is possible that SLP graduates may not have any direct clinical observation or hands-on experience or have received adequate post-basic training to cater to the needs of specific patient populations, such as individuals with PD.

## Aims of the study

Given the increasing numbers of PD and the high prevalence of individuals with motor speech and language impairments, it is necessary that SLPs be prepared to manage the assessment and treatment of patients with PD. The aim of this study is to investigate the perspective of SLPs working with PD in Malaysia, with primary outcomes of self-perceived competence in evaluating and managing several domains of PD and perceived barriers to management. The specific aims are the following:

1. Description of SLPs’ self-perceived competence in *evaluating* and *managing* the motor speech, language, oro-motor, cognitive and swallowing domains in patients with PD.
2. Investigation of differences in self-perceived competence within domains (motor speech, language, oro-motor, cognitive, swallowing).
3. Investigation of the influence of demographic factors and experience on overall self-perceived confidence.
4. Describe the most common barriers that Malaysian SLPs perceive when managing patients with PD in clinical practice.

The secondary outcomes of the study are the observation of current service delivery patterns (frequency of PD referrals, stage at referral, caseload sizes), intervention approaches used (behavioural vs Augmentative and Alternative Communication, AAC), multidisciplinary consultation patterns and confidence levels in managing rehabilitation risks associated with PD.

PD management is one of the mandatory courses for SLP certification in the country, and SLPs are expected to have received some disorder-specific training. However, certification is granted on completion of 300 clinical hours without any specifications for each disorder, and SLPs in lower- and middle-income countries cater to a wide range of patients in an environment of increasing specialisation, making it possible that SLP graduates have not had direct experience with individuals with PD. Moreover, since PD is still described and diagnosed as a primarily motor-based disorder in the literature, we expect the training to have been largely devoted to the management of its speech and motor disorders.

### Methodology

The study uses a survey questionnaire to collect self-reported quantitative data regarding the demographics of a sample of Malaysian SLPs working with patients with PD, their training and knowledge on topics related to PD, current practices and a self-evaluation of competence in assessing and managing several areas of PD assistance, including the barriers faced in this practice. Inferential statistics were run to establish any differences in the self-perceived competence between the areas of assessment and management of PD and the potential role of demographic and training factors.

### Study design

This study employed a cross-sectional design with data collected during March-December 2024 in Malaysia among practicing SLPs who work in public and private healthcare settings.

### Setting

SLPs in Malaysia who were actively practicing in various healthcare settings, including public and private clinics and hospitals, were targeted.

### Data collection

The survey was administered in English through Google Form. A poster was designed to enable sharing of study details and the questionnaire link. Participants were invited via email and WhatsApp messenger. These contacts were obtained from the Speech-Language Therapists Association of Malaysia (SPEAK), and snowball sampling was encouraged to recruit additional SLPs through other social networks.

Participants were required to provide their consent for data collection and processing at the beginning of the survey. The survey took approximately 5–10 min to complete and the collected data was transferred to Excel. To prevent multiple participation from the same participant, responses from the Google Forms survey were downloaded as a dataset and manually screened for duplicate entries by cross-checking participant demographics, timestamps and response patterns to ensure data integrity and prevent multiple participation from the same individuals. The data were collected between March and May 2023. This study was approved by the university ethics committee (JEP-2023-050). The data were only accessible by researchers. To ensure anonymity, no identifying information was collected, and data were protected.

### Measures

The survey used in this study is an adaptation of Niharika *et al*,^33^ a survey investigating PD management in SLPs working in India, offering a comprehensive investigation of self-perceived competence and barriers faced by SLPs working with PD in the country. Three experienced Malaysian SLPs adapted the questionnaire for the Malaysian context, making minimal adjustments on the demographic variables and the potential barriers. They further checked the survey questions for clarity and grammatical correctness to ensure its construct validity. A pilot survey with ten subjects further validated the questionnaire’s effectiveness, providing feedback for final adjustments before full implementation in the study. Data were stored on a secure, password-protected server accessible only to members of the research team.

The survey (online supplemental appendix A) comprised five sections, namely: *demographics* (seven closed questions)*, risk management* (one question on a 5-point Likert scale), *background knowledge on PD* (11 closed questions)*, current practices* (seven closed questions)*, self-perceived competence* (two questions on a 5-point Likert scale)*, barriers* (one multiple-choice question), for a total of 29 questions. The two questions on self-perceived competence were divided into five domains: speech, language, oro-motor skills, cognition and swallowing. Demographics and background knowledge questions serve the purpose of identifying the respondents in terms of their exposure to patients with PD and their training and knowledge on the subject, to inform our interpretation of results regarding confidence levels and barriers. Current practices and risk management questions informed the description of current service delivery patterns (frequency of PD referrals, stage at referral, caseload size), intervention approaches used (behavioural vs AAC), multidisciplinary consultation patterns, and confidence levels in managing rehabilitation risks associated with PD.

### Participants

Participants were certified SLPs working in Malaysia at the time of testing. To be included in the final analyses, SLPs had to have at least one active case of PD in their caseload. This led to the exclusion of two participants who did not have patients with PD in their caseload.

### Analyses

Since all survey questions were mandatory, the dataset contained no missing data. Inferential analyses were carried out on R V.4.3.1.^41^ First, a descriptive interpretation of the most common answers to the confidence questions is provided. For aim 2, pairwise differences between confidence levels around assessment and around management of the different areas of possible intervention were examined with a Wilcoxon signed-rank test, with a Bonferroni correction applied to adjust for multiple comparisons (k=10), resulting in a corrected significance threshold of α=0.005. For aim 3, a multiple regression model was run to examine whether demographic and training characteristics of the respondents predicted confidence levels. Regression analyses were conducted to explore associations between clinician characteristics and confidence scores, rather than to establish causal relationships. Since the analyses were primarily exploratory and descriptive in nature, exploratory factor analysis (EFA) was used to identify latent dimensions underlying confidence ratings, rather than to test a priori hypotheses or make population-level inferences. The analysis yielded two composite scores: MR1 for speech, language, and cognition confidence and MR2 for swallowing/oro-motor confidence. Factor score adequacy was evaluated to ensure reliability for subsequent analyses. Finally, multiple linear regression models were fitted separately for MR1 and MR2 to examine whether clinician characteristics predicted confidence in these domains. After a variance inflation factor to check for collinearity, the following predictors were kept: education, years of experience, work setting, PD knowledge, referral practices, referral stage and specialised training. All variables except years of experience and PD knowledge were factors, PD knowledge was a continuous variable obtained by summarising the number of correct responses in the questions around PD per participant. Aims 1 and 4 are summarised using response frequencies.

## Results

54 respondents (mean age 34, SD 5.69, range 25–47) completed the survey. This sample represents approximately 14% of the estimated 380 practising SLPs in Malaysia. However, as only SLPs with at least one active PD case were eligible to participate, and data on how many Malaysian SLPs work with neurological populations is unavailable, the true response rate among eligible participants cannot be precisely calculated.

Table 1 provides the demographic information of the participants. Respondents were practising in 14 different states and represented all regions of Malaysia, including Wilayah Persekutuan (except for Labuan), with 25.6% of them working in Kuala Lumpur.

**Table 1:** Demographic characteristics of participating SLPs (n=54)

Demographic variable	N (%)
Gender	
Female	50 (92.5)
Male	4 (7.5)
Education	
Bachelor’s	45 (83.3)
Master’s	9 (16.6)
Years of experience as SLP	
0–5	15 (27.7)
6–10	18 (33.3)
11–15	8 (14.8)
>15	13 (24)
Primary work setting	
Academic	2 (3.7)
Hospital (government)	46 (85.1)
Hospital (private)	4 (7.4)
Private clinic	0
Home therapy	1 (1.8)
Multiple	1 (1.8)
Specialised training on PD	
Yes	8 (14.8)
No	46 (85.1)

A summary of participants’ gender, age range, years of experience, workplace settings and caseloads.

PD, Parkinson’s disease; SLP, speech and language pathologist.

**Figure 1:**
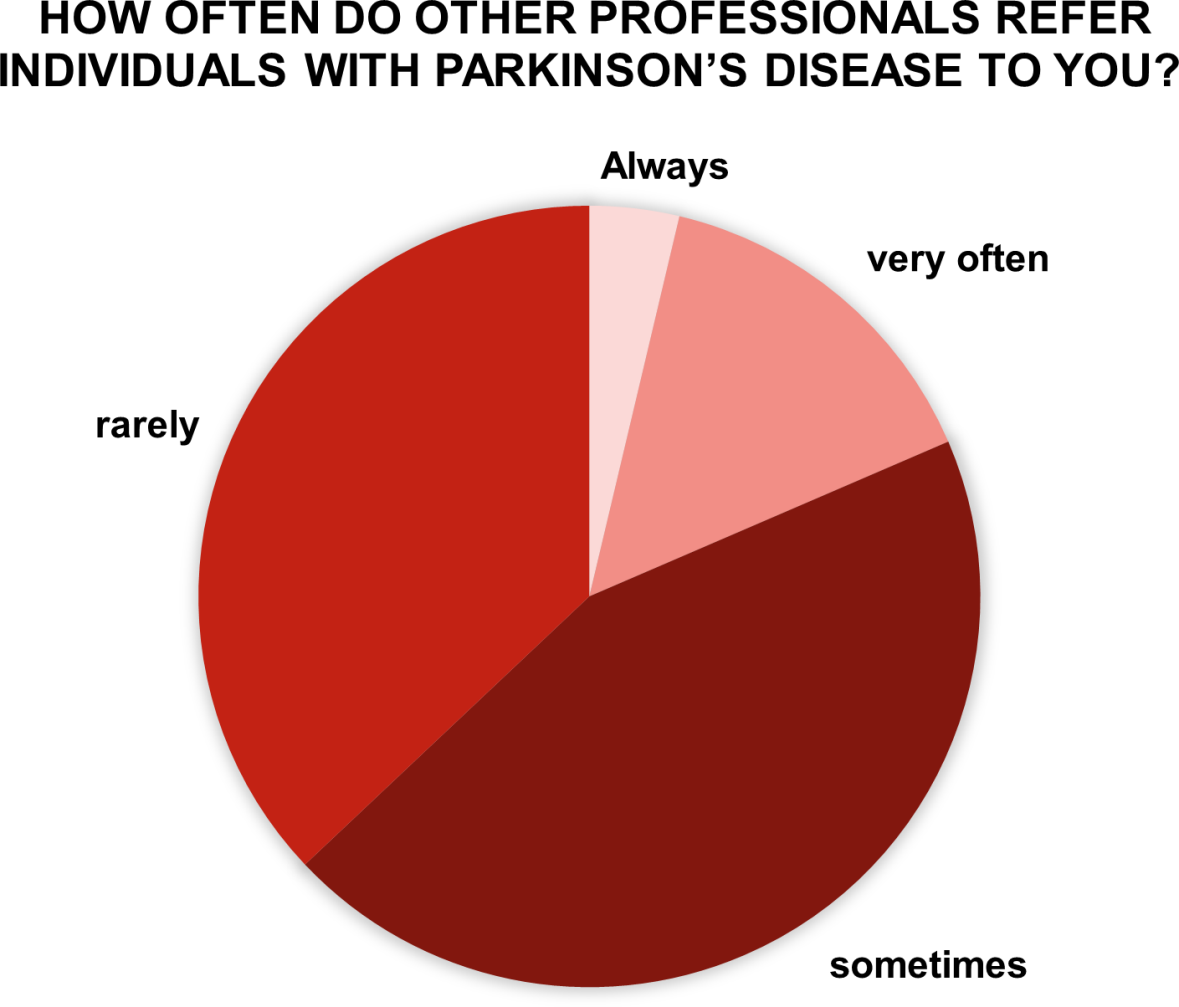
A pie chart illustrating the percentage distribution of patient with PD referrals to SLPs by other professionals. PD, Parkinson’s disease; SLP, speech and language pathologist.

Most participants declared having 1–5 patients with PD in their active caseload, who were referred at a middle or advanced stage of the disease. As shown in figure 1, around half of the participants are sometimes referred to patients with PD, 37% rarely are, and only under 20% are referred to always or very often. Interestingly, almost 90% of participants used AAC approaches only or in combination with behavioural approaches. Full results on section ‘background knowledge’ are provided in online supplemental appendix B, table 1.

Regarding knowledge of PD (pass/fail closed questions), while some questions had lower accuracy, overall accuracy percentages show good knowledge (73% accuracy across questions). Full results on ‘current practices’ are provided in online supplemental appendix B, table 2.

### Self-perceived competence

Participants were asked about their self-perceived competence when *assessing* and *managing* the following aspects in patients with PD: speech, language, oro-motor skills, cognition, and swallowing. Table 2 reports the results of this section. Overall, over 70% of participants considered themselves competent or highly competent in *assessing* swallowing and oro-motor skills, and over 60% in assessing speech and language. In contrast, only around 35% of them declared they felt competent or highly competent in assessing cognition, with almost half of them remaining neutral. Self-perceived competence in *managing skills* was overall lower than self-perceived competence in *assessment*. Around 53–57% of participants are competent to highly competent in managing swallowing and oro-motor skills, and around 43% of them are in managing language and speech. Participants are once again the least competent in managing cognition. Participants were also asked about their confidence in managing the risks involved in PD rehabilitation. Just over half of the participants declared they were confident to highly confident, with around 33% feeling somewhat confident (online supplemental appendix B, table 3). The question was removed from the inferential analyses given its general scope, not pertaining to SLP.

**Table 2:** SLPs’ self-perceived competence in assessing and managing patients with PD in different areas of SLP practice, from not competent to highly competent (n=54)

Self-perceived competence assessing	Incompetent	Somewhat competent	Neutral	Competent	Highly competent
Speech	2 (3.7%)	5 (9.3%)	14 (25.9%)	27 (50%)	6 (11.1%)
Language	0	7 (12.9%)	11 (20.3%)	27 (50%)	9 (16.6%)
Oro-motor skills	2 (3.7%)	5 (9.3%)	9 (16.6%)	28 (51.8%)	10 (18.5%)
Cognition	0	10 (18.5%)	25 (46.2%)	14 (25.9%)	5 (9.3%)
Swallowing	3 (5.5%)	5 (9.3%)	6 (11.1%)	26 (48.1%)	14 (25.9%)
**Self-perceived competence managing**					
Speech	1 (1.8%)	6 (11.1%)	17 (31.4%)	24 (25.9%)	6 (11.1%)
Language	0	6 (11.1%)	17 (31.4%)	24 (25.9%)	7 (12.9%)
Oro-motor skills	0	5 (9.3%)	20 (37%)	19 (35%)	10 (18.5%)
Cognition	1 (1.8%)	8 (14.8%)	28 (51.8%)	13 (24%)	4 (7.4%)
Swallowing	1 (1.8%)	6 (11.1%)	12 (22.2%)	22 (40.7%)	13 (24%)

PD, Parkinson’s disease; SLP, speech and language pathologist.

Pairwise Wilcoxon signed-rank tests were conducted to examine differences in confidence ratings across domains. For self-reported confidence in assessing, clinicians reported significantly lower confidence in assessing cognition compared with speech (p=0.027), language (p=0.001), oro-motor (p=0.002) and swallowing (p=0.012). No significant differences were observed among speech, language, oro-motor, and swallowing. For self-perceived confidence around managing tasks, confidence in managing cognition was significantly lower than speech (p=0.032), language (p=0.004), oro-motor (p=0.012) and swallowing (p=0.010). Other pairwise comparisons did not reach significance after correction. The domain of cognition is therefore consistently rated significantly lower than other domains in both assessment and management contexts.

Next, regression analyses were conducted to examine whether demographic and training variables predicted confidence in the composite scores MR1 (cognition/language/speech) or MR2 (swallowing/oro-motor). None of the predictors significantly explained variance in MR1 or MR2 (all ps>0.20), and adjusted R^2^ values were low (<0.10), suggesting confidence differences reflect domain-specific patterns rather than being explained by other factors.

### Barriers

**Figure 2:**
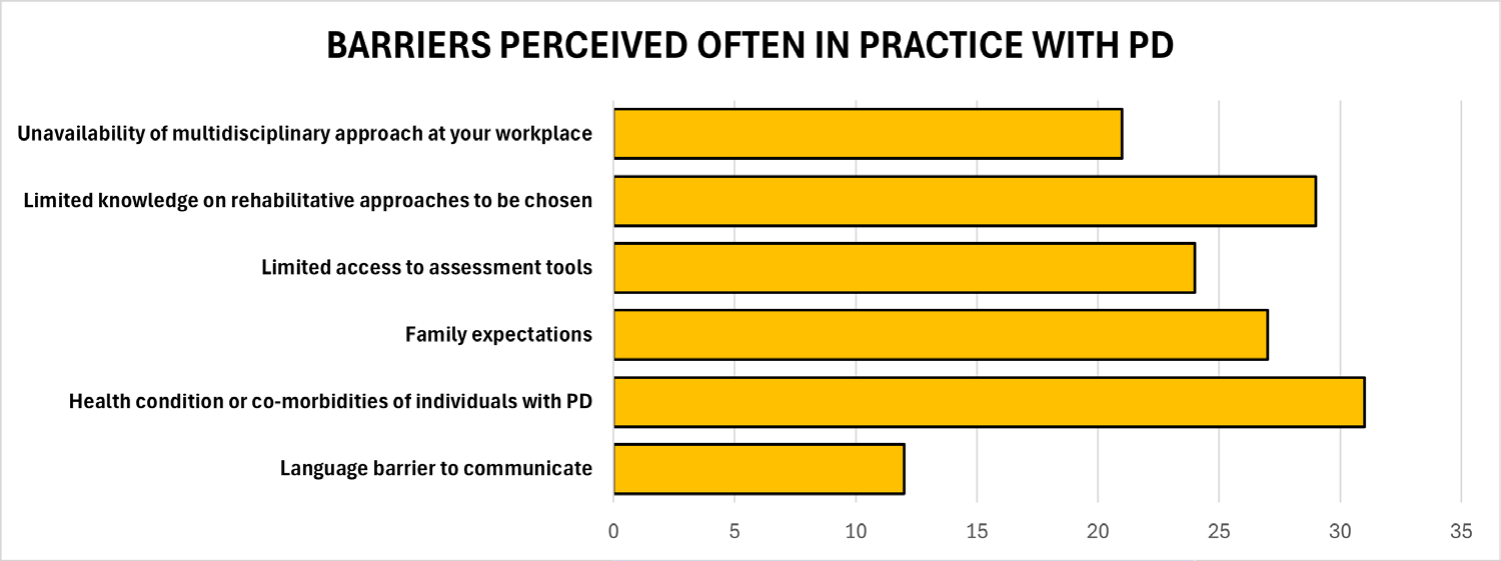
Barriers perceived often with practice with PD. A bar chart illustrating number of times each barrier was selected (n=144). PD, Parkinson’s disease.

In a multiple-choice question, participants were asked to select which barriers they perceived often in their practice with individuals with PD. Participants could select more than one answer. Total number of answers recorded is 144. Results are reported in figure 2. The most frequently perceived barrier by SLPs when managing people with PD was the health condition of the patient and/or comorbidities (31 selections, 21.5% of answers), followed by limited knowledge around the rehabilitative approaches to be chosen (29 selections, 20%). The barrier encountered the least was the language barrier to communicate (12 selections, 8%).

## Discussion

This study represents an exploratory initial investigation of Malaysian SLPs’ knowledge, self-perceived competence and barriers in the treatment and management of patients with PD. Overall, the majority of participants reported being competent or highly competent in evaluating and treating swallowing, oro-motor skills and speech and language assessments. However, only a minority of them believed they were competent or highly competent with cognition. Furthermore, participants in the survey revealed that the barriers faced most often are the health conditions or comorbidities of the patients, limited knowledge on the appropriate rehabilitative approaches, limited access to assessment tools and family expectations. It is worth noting that this study is limited to SLP practice in Malaysia, and the findings cannot be generalised to other countries or global contexts.

### Malaysian SLPs feel less competent in evaluating cognitive function among PD

Cognitive impairment is a major predictor of negative outcomes in patients with PD, including a decline in functional capacity, increased caregiver burden and risk of dementia, but only a minority of patients with PD receive a comprehensive neuropsychological evaluation (38% in^42^), and receive any intervention (50% of those with mild cognitive impairment, ibid.). This indicates a significant gap between the need for cognitive assessment and its provision in the context of PD. Moreover, cognitive status highly correlates with functional communication. As a result, cognitive assessment is a crucial aspect of managing PD in speech language therapy.^22^

Malaysian SLPs in our data systematically show significantly lower confidence in their skill set assessing cognitive function in patients with PD compared with other areas of SLP intervention. In fact, nearly half of the respondents took a neutral stance. This finding is in contrast with results from the survey conducted in India, where most SLPs perceived themselves reported competent in the cognitive function assessment,^33^ while it aligns with results from the UK and Australia, revealing that only a limited number of British SLPs conduct cognitive and mood assessments^27^ and fewer than one-third of Australian SLPs had access to cognitive evaluations for individuals with progressive neurological disorders when managing cognitive-communication disorders.^30^ This is concerning since^43^ ’s recent study revealed the significant and complex care needs of those with cognitive impairment in PD, with a greater need for assistance observed in all seven of the evaluated daily tasks compared with those without significant cognitive impairment. Despite this, healthcare consultations were not more frequent for those with cognitive impairment, contrary to the researchers’ hypothesis. This study confirms this trend for Malaysia, with SLPs feeling confident in their skill set for most areas covered except for cognition. It is therefore strongly recommended that educators concentrate and provide future SLPs with more training in targeting cognitive function, given its importance for a comprehensive evaluation of the patient but also for treatments targeting cognitive functions to boost language, as proposed for PD in line with efficient treatments in aphasia.^16 21^

In our participants, self-perceived competence levels are not influenced by demographic factors or factors linked to current practices, a result that is potentially due to the low variability in our sample in these variables, limiting its generizability. In fact, the role of demographic factors on self-efficacy is well known, for example, Tilmon^44^ in Columbia found self-efficacy among SLPs in various settings to differ significantly among the four groups based on years of experience and^45^ showing that Irish SLPs with greater experience used a broader range of therapies and techniques across multiple variables. As for education level, SLPs with higher education levels were shown to face fewer barriers to EBP in Japan and Malaysia.^44^ These studies emphasise the importance of ongoing explicit education in EBP for SLPs to improve their service delivery.

### Malaysian SLPs focus more on oral-motor function

Malaysian SLPs tend to concentrate more on oral-motor function, where SLPs feel overall confident in their skills. Our study’s findings align with those of a UK-based investigation involving SLPs, which revealed that the majority of SLPs conducted oral motor examinations.^27^ Only a small proportion of SLPs in the UK used alternative formal assessments for intelligibility, voice, language or psychosocial impact. Surprisingly, despite the presence and impact of cognitive communication deficits, research focused on therapeutic interventions for motor speech disorders has remained the primary focus, with less attention given to cognitive-communication disorders.^35^ The discrepancy in the evidence bases for these two disorders likely resulted in a limited understanding of cognitive-communication disorders among SLPs, necessitating further research in this area.

In the domain of swallowing, a study conducted in 2015^36^ found that the majority of Malaysian SLPs surveyed did not feel competent or confident in providing dysphagia services, primarily due to insufficient training and workplace infrastructure. Another significant factor identified was the lack of awareness and collaboration of other Malaysian healthcare professionals in managing dysphagia and the absence of a multidisciplinary team approach to dysphagia management.^36^ However, our study’s results appear to demonstrate a positive shift in this area, as 70% of our respondents reported feeling competent in assessing and treating swallowing disorders. This trend signifies a potential evolution towards a more cohesive and multidisciplinary approach to dysphagia management within the Malaysian healthcare system. Contrarily, a similar study conducted in India found that SLPs in India had relatively lower competence in evaluating and managing swallowing aspects of PD.^33^ The authors of this study suggest that this disparity could be attributed to the variability in dysphagia practice patterns among SLPs in India, with barriers related to access to instrumental evaluations and limited clinical education and training.^46^

### Navigating barriers in PD management: insights and strategies for effective care

The participants in the study noted that the lack of evidence and guidelines on rehabilitative approaches was a significant hindrance to incorporating EBP into clinical settings, raising concerns about the quality of care. According to Swales *et al*,^30^ 42% of SLPs who provided cognitive communication services for individuals with PD and completed the survey indicated a dearth of evidence on managing cognitive communication changes. Another investigation by Swales *et al*^32^ uncovered that although most individuals with PD who received SLP services were satisfied with their support, several issues were identified, including constraints on the number of services received, clinicians’ knowledge of PD and management options. A study examining the present practices of Malaysian SLPs working with dysarthria discovered that EBP training was not formally included in the Malaysian SLPs’ curriculum.^47^ Since professional guidelines and other evidence statements can impact clinical decision-making, SLPs face the challenge of incorporating EBP training with no formal guidelines for managing dysarthria or other speech and language disorders^43^. Even when research exists on a particular treatment approach, there may still be obstacles to its implementation in clinical practice. Some SLPs in Columbia reported feeling uncertain about how to apply research findings to clinical practice due to discrepancies between studies and real-world clinical practice.^47^

Our participants reported that the most common obstacle in working with individuals with PD was the patient’s health condition and any accompanying comorbidity. People with PD often experience mobility issues, difficulties with daily activities, emotional fluctuations, cognitive impairments, communication challenges and physical discomfort, as indicated by the Parkinson’s Disease Questionnaire-39 (PDQ-39).^48^ A study in India found that although SLPs reported feeling competent in assessing and managing patients with PD, health conditions or comorbidities, family expectations regarding therapy outcomes and the lack of a multidisciplinary approach were reported as major barriers faced by SLPs.^33^ Since mobility worsens progressively in PD and most SLP services are provided in clinics, providing services in the patients’ homes would alleviate their dependence on others and serve as a barrier to accessing SLP services.^32^ A preliminary study in Malaysia also showed that delivering intensive voice therapy to individuals with PD via smartphone videoconferencing is feasible, with good treatment attendance, measurable gains in vocal loudness and intelligibility and favourable patient-reported outcomes, underscoring the need for future studies to systematically evaluate and expand telepractice-based SLP services for this population.^49^

It is interesting to note that, despite the prevalence of multilingualism adding a layer of complexity to healthcare delivery compared with more monolingual countries, Malaysian SLPs in our survey do not perceive language barriers as particularly problematic, accounting for only 22% of the total. Malaysia is a highly multilingual country with Malay as the national language, English as a widely used second language and large Mandarin, Tamil and indigenous language communities distributed across Peninsular Malaysia and East Malaysia. In routine clinical practice, Malaysian SLPs typically prioritise assessment and treatment in the patient’s functionally dominant language, draw on available Malay-based and English-based tools, and supplement these with culturally and linguistically adapted informal or dynamic assessments when standardised tests are unavailable for a given language or dialect. This finding is in contrast with previous research from North America, where language barriers substantially impacted the work of SLPs, physical therapists and occupational therapists.^50 51^ On the other hand, it confirms results from a survey on SLP practices with multilingual children in Malaysia and other Southeast Asian countries,^39^ suggesting that Malaysian SLPs are highly confident in managing multilingual caseloads despite the challenges it poses due to a lack of appropriate tools in the target languages and limited training on specific matters of multilingual language. Addressing these challenges is still of paramount importance in order to ensure equitable access to quality healthcare services. Strategies such as providing language training for healthcare professionals, employing interpreters or bilingual staff and using technology-based translation tools can all contribute to overcoming these obstacles and fostering effective communication in healthcare delivery.

## Limitations

This study has several limitations that should be acknowledged. First, the generalisability of study findings is limited to a snapshot specific to Malaysian SLPs. While our sample of 54 SLPs represents approximately 14% of the estimated 380 practising SLPs in Malaysia, there was little variation in the distribution of demographic variables, with most SLPs being females working in government settings, a low number of patients with PD in their active caseloads and no specific training, masking potential effects of these factors on self-perceived competence. Moreover, a formal sample size calculation was not conducted prior to deploying the survey, and the cross-sectional design limits interpretation to correlations rather than causality. Future studies should address whether experience-related differences reflect accumulated practice, additional training or cohort effects in education with longitudinal designs. Additionally, the reliance on self-reported data may introduce response bias, including social desirability bias, which may be introduced when the participants become aware that this study was led by experts in the same field.

### Strengths of the study

This is the first study in Malaysia to examine SLPs’ knowledge, self-perceived competence and perceived barriers in assessing and managing people with PD, addressing a previously unexplored aspect of service delivery in this context and providing clear evidence gap in low- and middle-income settings. The use of a nation-wide sample recruited via the professional association and snowballing captures real-world practices across service contexts and includes only clinicians with active PD caseloads, enhancing clinical relevance. The survey was structured around key SLP domains (speech, language, oro-motor skills, cognition and swallowing), enabling domain-specific profiling of perceived competence and targeted training implications.

### Lessons for practice

1. The observed lack of confidence among SLPs in evaluating the cognitive abilities of patients with PD highlights the need for enhanced and specific educational initiatives that emphasise cognitive assessment techniques and intervention approaches.
2. To provide effective care for patients with PD, SLPs should work closely with other healthcare professionals, addressing the importance of an interdisciplinary approach considering the patient’s overall health conditions and potential language barriers.
3. Future studies should focus on a more comprehensive investigation of the specific barriers, such as language differences, that SLPs encounter in managing PD to develop strategies to mitigate these barriers and enhance service delivery effectively.

## Supplementary material

10.1136/bmjopen-2025-106250 online supplemental file 1

## Data Availability

Data are available upon reasonable request.
