# Supplementary material for: Catering to patients with Parkinson’s disease: a survey on self-perceived competence and barriers of speech and language pathologists in Malaysia
Source: BMJ Open. 2026 Jun 9;16(6):e106250. doi: 10.1136/bmjopen-2025-106250 (PMC13264918; doi:10.1136/bmjopen-2025-106250)
Supplement: online supplemental file 1 [file bmjopen-16-6-s001.docx]

**Appendix A: Survey for Speech-Language Therapists’ (SLTs’) Self-Perceived Competence in Serving Individuals with Parkinson's Disease’ (IWPD) in Malaysia**

**Demographic Details**

1. Age: _______________________

2. Gender:

🞏Female 🞏Male

3. Education:

🞏Undergraduate (Bachelor's degree) 🞏Postgraduate (Master's degree) 🞏Postgraduate (PhD)

4. State of current practice:

🞏Johor 🞏Kedah 🞏Kelantan 🞏Melaka 🞏Negeri Sembilan 🞏Pahang 🞏Penang 🞏Perak 🞏Perlis 🞏Sabah 🞏Sarawak 🞏Selangor 🞏Terengganu 🞏Kuala Lumpur 🞏Labuan 🞏Putrajaya

5. Total work experience in the field of speech-language pathology (years):

🞏0-5 🞏6-10 🞏11-15 🞏>15

6. Primary work setting:

🞏Hospital (government) 🞏Hospital (private) 🞏Private clinic 🞏Private practice 🞏Academic institutions 🞏Non-profit organizations 🞏Home therapy (self-funded) 🞏Telepractice (self-funded) 🞏Multiple work settings

7. Have you received specialized training to work with individuals with Parkinson’s disease?

🞏Yes 🞏No

**Knowledge on PD**

8. Which of the following domains are affected in individuals with Parkinson’s disease? 🞏Speech 🞏Language 🞏Cognition 🞏Swallowing 🞏All of the above (correct)

9. The pattern of speech changes associated with Parkinson’s disease is:

🞏Hypokinetic dysarthria (correct) 🞏Hyperkinetic dysarthria 🞏Spastic dysarthria 🞏Flaccid dysarthria

10. At what stage of Parkinson’s disease can speech changes be identified?

🞏Early stage (correct) 🞏Middle stage 🞏Advanced stage

11. Which is the neurotransmitter that is linked with the pathophysiology of Parkinson’s disease?

🞏Acetylcholine 🞏Dopamine (correct) 🞏GABA 🞏None of the above

12. A change in handwriting which is seen in individuals with Parkinson’s disease is called: 🞏Micrographia (correct) 🞏Macrographia 🞏Agraphia 🞏All of the above

13. The most common medication prescribed for individuals with Parkinson’s disease is: 🞏Levodopa (correct) 🞏Carbamazepine 🞏Pyridoxine 🞏None of the above

14. Parkinson’s disease is a/an:

🞏Auto-immune disease 🞏Neuro-developmental disorder 🞏Metabolic disorder 🞏Neuro-degenerative disorder (correct)

15. Parkinson’s disease is caused by the dysfunction of:

🞏Substantia Nigra (correct) 🞏Putamen 🞏Globus Pallidus 🞏None of the above

16. Which of the following is NOT a motor symptom of Parkinson’s disease?

🞏Tremor 🞏Bradykinesia 🞏Flaccid muscles (correct) 🞏Freezing

17. What type of tremor is usually seen in individuals with Parkinson’s disease?

🞏Essential Tremor 🞏Holmes tremor 🞏Dystonic tremor 🞏Pill rolling Tremor (correct)

18. What is the most recommended voice therapy technique used for individuals with Parkinson’s disease?

🞏Resonant Voice Therapy 🞏Lee Silverman Voice Therapy (correct) 🞏Semi-occluded Voice Therapy 🞏Vocal Function Exercise

**Current practices**

19. Number of individuals with Parkinson’s disease in your active caseload per month:

🞏1-5 cases 🞏6-10 cases 🞏11-20 cases 🞏>20 cases 🞏None

20. How often do other professionals refer individuals with Parkinson’s disease to you?

🞏Always 🞏Very often 🞏Sometimes 🞏Rarely 🞏Never

21. From which of these professionals do you mostly receive referrals?

🞏Neurologist 🞏Physician 🞏Geriatrician 🞏Patients themselves report to you 🞏None of the above

22. At what stage will individuals with Parkinson’s disease be referred to you?

🞏Early stage 🞏Middle stage 🞏Advanced stage 🞏I receive no referral

23. Whom do you often consult or discuss while working with individuals with Parkinson’s disease?

🞏Neurologist 🞏Dietician 🞏Another Speech-Language Therapist 🞏Physiotherapist 🞏Psychologist 🞏Psychiatrist 🞏I do not consult

24. Which of the following do you mostly opt for in the rehabilitation of individuals with Parkinson’s disease?

🞏Behavioral intervention 🞏Augmentative and Alternative Communication (AAC) 🞏Combination of behavioral and AAC

25. How do you update yourself about Parkinson’s disease?

🞏Books 🞏Journal articles 🞏Webinars 🞏Conferences 🞏Certified courses

**Perceived competence and confidence**

26. Rate your self-perceived competence in assessing each of the domains in individuals with Parkinson’s disease:

Speech | Language | Oro-motor skills | Cognition | Swallowing (5 - Highly competent | 4 - Competent | 3 - Neutral | 2 - Somewhat incompetent | 1 - Incompetent)

27. Rate your self-perceived competence in managing each of the domains in individuals with Parkinson’s disease:

Speech | Language | Oro-motor skills | Cognition | Swallowing (5 - Highly competent | 4 - Competent | 3 - Neutral | 2 - Somewhat incompetent | 1 - Incompetent)

28. How confident are you to manage the risk involved in the rehabilitation of people with Parkinson’s disease?

🞏Completely confident 🞏Fairly confident 🞏Somewhat confident 🞏Slightly confident 🞏Not confident at all

**Challenges**

29. Which of these often acts as a barrier for your practice with individuals with Parkinson’s disease?

🞏Language barrier to communicate

🞏Health condition or co-morbidities of individuals with Parkinson’s disease

🞏Family expectations

🞏Limited access to assessment tools

🞏Limited knowledge on rehabilitative approaches to be chosen

🞏Unavailability of multidisciplinary approach at your workplace

Appendix B

| **Current practices with PD** | N(%) |
| --- | --- |
| *Number of individuals with PD in active caseload per month*  1-5  6-10  11-20  >20 | 43 (79.6%)  6 (11.1%)  2 (3.7%)  3 (5.5%) |
| *Stage of PD at time of referral*  No referral  Early  Middle  advanced | 2 (3.7%)  9 (16.6%)  31 (57.4%)  12 (22.2%) |
| *Type of intervention used in rehabilitation of PD*  Behavioural intervention  Augmentative and Alternative Communication (AAC)  Combination of behavioural and AAC | 6 (11.1%)  6 (11.1%)  42 (77.7%) |
| *Other professionals consulted for PD cases*  Neurologist  Dietician  Another Speech-Language Therapist  Physiotherapist  Psychologist/Psychiatrist | 29 (54%%)  20 (37%%)  54 (95%)  19 (35%)  8 (15%) |

**Table 1.** Speech and Language Pathologists’ perceptions of patient awareness and help-seeking behaviours

| Which of the following domains are affected in individuals with Parkinson’s disease? | 42 (78%) |
| --- | --- |
| The pattern of speech changes associated with Parkinson’s disease is | 46 (85%) |
| At what stage of Parkinson’s disease can speech changes be identified? | 20 (37%) |
| Which is the neurotransmitter that is linked with the pathophysiology of Parkinson’s disease? | 44 (81%) |
| A change in handwriting which is seen in individuals with Parkinson’s disease is called | 35 (65%) |
| The most common medication prescribed for individuals with Parkinson’s disease is | 50 (92.5%) |
| Parkinson’s disease is a/an | 54 (100%) |
| Parkinson’s disease is caused by the dysfunction of | 41 (76%) |
| Which of the following is NOT a motor symptom of Parkinson’s disease? | 32 (59%) |
| What type of tremor is usually seen in individuals with Parkinson’s disease? | 22 (41%) |
| What is the most recommended voice therapy technique used for individuals with Parkinson’s disease? | 51 (94%) |

Table 2. Number and percentage of correct answers on the background knowledge section of the survey.

|  | Not confident at all | Slightly confident | Somewhat confident | Fairly confident | Completely confident |
| --- | --- | --- | --- | --- | --- |
| Confidence managing the risk involved in the rehabilitation of PD | 1 (1.8%) | 4 (7.4%) | 18 (33.3%) | 24 (44.4%) | 7 (12.9%) |

**Table 3.** **Confidence Managing the Risk Involved in the Rehabilitation of PD**

*Response to a 5-point Likert scale survey, presenting the numbers of SLPs in each category and the corresponding percentage relative to the total sample size*
