# Supplementary material for: Catering to patients with Parkinson’s disease: a survey on self-perceived competence and barriers of speech and language pathologists in Malaysia
Source: BMJ Open. 2026 Jun 9;16(6):e106250. doi: 10.1136/bmjopen-2025-106250 (PMC13264918; doi:10.1136/bmjopen-2025-106250)
Supplement: Supplementary file 2 [file bmjopen-2025-106250.reviewer_comments.pdf]

PEER REVIEW HISTORY

BMJ Open publishes all reviews undertaken for accepted manuscripts. Reviewers are asked to complete a checklist review form and are provided with free text boxes to elaborate on their assessment. These free text comments are reproduced below.

ARTICLE DETAILS

Title (Provisional)

Catering to patients with Parkinson's disease: A survey on self-perceived competence and barriers of Speech and Language Pathologists in Malaysia

Authors

Chu, Shin Ying; Kamarulzaman, Nursabrina Binti; Siau, Ching Sin; Woo, Pei Jun; Zhang, Mike Minwen; Yuen, Man-Ching; Garraffa, Maria; Smith, Giuditta

---

VERSION 1 - REVIEW

**Reviewer**	**1**
**Name**	**McAuliffe, Megan**
**Affiliation**	**University of Canterbury**
**Date**	**18-Aug-2025**
**COI**	**None**

Thank you for the opportunity to review this interesting research examining the self-described competence of SLPs working in Malaysia and the challenges they face. The method included an adaptation of an existing published survey and was generally well described.

I have several comments that I believe would enhance the work:

(a) The aims could have been more clearly articulated and related directly to the analyses undertaken in the results section. I'd suggest these be framed as, for example: (1) What are the current practices of SLPs in Malaysia in relation to the care of people with PD? (2) What are their self-perceived levels of competence and barriers to management? and (3) What is the relationship between SLPs self-perceived confidence and their levels of experience and knowledge?

(b) I was surprised to see the large number of correlations that were run to examine the relationship between confidence/competence results and a range of demographic factors. As mentioned in my comment about the aims, this wasn't foreshadowed in the aims.

(c) Results: There were a considerable number of correlations run, but I did not see any corrections for multiple comparisons. The risk of Type I error was likely high. Several of the predictor variables (e.g., age, years of experience, education) were also likely highly correlated and there was no check

for multicollinearity. I'd suggest that this is undertaken and further consideration be given to the variables included in the correlations.

As an aside, I was interested to read of the high proportion of SLPs that used AAC or a combination of behavioural and AAC approaches with their clients. This seemed a relatively high reported use of AAC compared with clinical practice in my own and nearby countries. I was interested to know more about what that entailed and why it was used.

Overall, these data will be useful for clinical training and professional development for SLPs in Malaysia. I found the results interesting and appreciated the opportunity to review the paper.

**Reviewer**	**2**
**Name**	**Johansson, Kerstin**
**Affiliation**	**Karolinska Institute**
**Date**	**21-Aug-2025**
**COI**	**None**

Thank you for the possibility to take part of your study where SLPs' have self-rated their knowledge of, and challenges in, SLP clinical practice to patients with Parkinson's disease in Malaysia!

The SLPs' responses to the two central questions in the 29-item survey regarding self-perceived competence show that approximately 60-70% of the respondents feel competent in assessing and treating oro-motor skills, speech, language and swallowing. In contrast, only 35% feel competent in managing cognition. Common barriers to clinical practice include the patients' health conditions, family expectations, and limited knowledge of rehabilitation approaches. Additionally, a smaller part, around 40%, of respondents reported limited access to assessment tools and the unavailability of a multidisciplinary team as barriers.

In healthcare, the evidence base for assessment and treatment is constantly evolving. All levels–governmental, organizational, professional and individual clinicians–must integrate new knowledge and update practice when necessary. Clinicians are responsible for continuously evaluating the content of their clinical work to ensure it best meets the needs of the specific patient groups. Therefore, valid evaluations of SLPs' knowledge (and gaps) and service content can provide essential insights for the development of the SLP profession and practice in a given country. These evaluations can also inform educational programs about areas that may need expansion to improve SLPs' competencies.

This investigation can serve as a valuable foundation for developing SLP practices for patients with Parkinson's disease in Malaysia. However, in my view, the findings may be of more limited interest to an international audience.

Some comments on the manuscript:

Abstract:

1. In the conclusion and elsewhere, the manuscript states "Malysian SLPs are competent in x". Consider using "feel competent", as this better reflects what is investigated. Also, responses to

background questions suggest varying levels of knowledge: only 37% know at what disease stage speech changes can be detected, and 65% are familiar with micrografia.

2. In “Strengths and limitations” the manuscript mentions “use of a qualitative design, ... provided rich insights”. However, the study is quantitative. While qualitative methods could indeed have provided rich insights, the short (5-10 minutes to complete) survey with only two central questions offers more superficial–though still valuable–information.

3. In “Primary... outcomes” the stated goals do not align with the actual investigation. The outcomes mention identifying key themes and gathering recommendations, which suggests a qualitative approach. The study instead reports quantitative self-ratings.

Methods:

1. Motivation for the choice of survey questions is missing. It’s great to adapt a survey from another study, but still some reasoning and references about content validity are needed. The inclusion of the seven questions on Parkinson’s disease knowledge are not clearly explained–what does answering these correctly indicate about the respondents’ knowledge?

2. Recruiting via a professional association and snowball sampling seems relevant. Were any reminders sent? Why not also via social media SLP groups? To assess how representative the results are, it would help to know approximately how many of the 380 SLPs in Malysia work with neurological patients.

Results:

1. Titles of Figure 1, Table 2 and Table 3 are unclear and do not match the content. For example:
   - Table 2 is described as showing “SLPs’ perceptions of patient awareness and help-seeking behaviours”, but it present “Current practices with PD”
   - Table 3 is said to show “SLPs’ perceived confidence in assessing patients with PD...” but it presents the SLPs’ ratings on how competent they feel.

2. The description of the analysis should be placed at the end of the Methods section. Table 5 “Inferential statistics...” could be moved to the supplementary material. Main results should be summarized in the Results section, and statistical reporting results should follow the journal’s format.

3. Table 4 refers to “Confidence managing the risk involved in the rehab of PD”. What are these risks?

4. Figure and table titles are usually placed **above** the respective figure/table.

Discussion:

1. The discussion on multilingualism is relevant, but more context about the situation in Malaysia would have been helpful, for example, number and distribution of languages, and how SLPs manage multilingualism in their practice, for example when it comes to language assessment and treatment.

2. SLP home services is mentioned, which may be beneficial for patients with Parkinson’s disease. Maybe also mention studies about SLP tele practice?

Manuscript:

1. "Terminology: “Challenges” and “barriers” are used interchangeably, consider choosing one of the terms. The same applies to “competency” and “confidence”.

2. Reference formatting is inconsistent. Only on page 6 is the “author, year” format used.
3. Several in-text references are missing in the reference list.
4. Reference list formatting varies.

**Reviewer**	**3**
**Name**	**Dack, Kyle**
**Affiliation**	**University of Bristol, MRC Integrative Epidemiology Unit**
**Date**	**29-Oct-2025**
**COI**	**None**

Statistical review summary:

This is an exploration of trends within the specific context of Malaysia. The analysis is fairly straightforward – describing the distribution of survey responses, and exploring correlations between variables. It seems to be that it is primarily a descriptive study – there is no particular exposure-outcome association being tested, and the interpretation does not intend to generalize beyond this population.

The editor may have guidance on this, but I suggest possibly stating in the title or abstract that it is a descriptive analysis to make it clear how results should be interpreted.

The primary issue with the manuscript is that it does not adhere to the STROBE reporting guidelines, there are important elements of the methodology not reported. I have highlighted what I feel are the most important omissions below, but generally speaking, all of the comments and queries can be resolved by applying the STROBE checklist and following the guidance provided in the elaboration document: https://pubmed.ncbi.nlm.nih.gov/25046751/

Where relevant I have added a STROBE reference (e.g. “[item 1]”) to help the authors locate further information if needed.

Detailed comments:

Abstract:

1. What is written is very clear. However, it isn’t immediately clear from the abstract alone whether the study is qualitative or quantitative. I suggest adding a section summarising the main statistical methods, and supporting the claimed results with statistical details.

Introduction

2. Clearly written and informative, the introduction provides the context and rationale for this study. As a minor suggestion, the aims section is long, and clarity could be improved by numbering specific aims.

Methods:

3. I suggest applying more subheadings, relevant to each item in the STROBE reporting guidelines. This will help readers navigate and ensure that everything is reported in a logical order. As a suggested example, this could be “study design”, “setting”, “participants”, “variables”, “statistical analysis”.

4. It would help readers if the methods began with a high-level overview of the study design, setting, dates, and any other key information. Currently it begins with details of participants and procedures but this is harder to follow without an overview of the design. [item 4]

5. Setting: I suggest adding more specifics of the setting so readers understand the context of the findings, e.g. where were participants working, what types of environment, how specifically was this setting targeted for recruitment? [item 5]

6. Table 1. This table is helpful but is more suited to the results section.

7. Variables: The second paragraph of the methods gives an overview of the survey from which variables were measured. But it would be helpful to know further how each of these relates to the aims, i.e. what was the purpose of measuring each category? For the key variables which the authors intend to analyse, it would be helpful to specify each item. Were any of these items combined or was each to be analysed separately? [item 7]

8. Statistical methods: This section does not meet STROBE reproducibility standards. Information should be provided so that readers can more or less predict what tables and figures they will see in the results, before viewing them. This aids readability and accurate interpretation by readers. I suggest adding a new paragraph or paragraphs, detailing (a) the software used (already reported), (b) that demographic characteristics were summarized (and how), (c) how practices of SLP with PD are summarized, (d) what comparisons or inferential statistics were generated – for which variables, how they relate to the aims, and the specific statistical tests.

Results:

9. In each figure and table it is helpful to remind readers of the sample size, particularly when percentages are reported such as in figure 1.

10. Table 3. I recommend adding the % sign to the percentages so these are not misinterpreted as anything else.

11. Inferential statistics: As there was no time interval between measuring the demographic characteristics and reported competence levels, it is not possible to determine cause and effect. Also because no modelling is performed to adjust for external factors which could influence both variables. I suggest reframing this part of the analysis as exploring correlations between variables, and removing reference to “influences”, “predicted” etc, as this language is not supported by the data type.

12. To align with STROBE reporting guidelines, please support the in-text results with statistics, for example: “Age and years of experience were the most significant factors, with older and more experienced participants feeling more competent in assessing all aspects and managing speech and

language as well as more competent in managing risks" report the mean or median confidence levels for the different age groups to demonstrate this.

13. Table 5. A footnote should explain what each statistic means, e.g. U, r, H(4). For some it is not really clear how a comparison was made or what the statistic represents, because the variables are not continuous – adding information in the methods would help clarify this.

Discussion

14. There are currently no strengths or limitations stated. I suggest the major limitation and caveats for the interpretation which should be noted are that (a) much of it is a descriptive analysis, and as such is specific to this population and (b) the comparisons which were made are all bivariate – no adjustment was made for external factors (confounding), and so the estimated correlations could be biased, and (c) only correlations can be determined, not causality, due to the lack of a temporal distinction between variables.

VERSION 1 - AUTHOR RESPONSE

Reviewer: 1

-The aims could have been more clearly articulated and related directly to the analyses undertaken in the results section. I'd suggest these be framed as, for example:
-What are the current practices of SLPs in Malaysia in relation to the care of people with PD?
-What are their self-perceived levels of competence and barriers to management?
-What is the relationship between SLPs self-perceived confidence and their levels of experience and knowledge?

Response: Thanks for your comment. Please fins this addressed in lines 133-141.

-**Results**: -I was surprised to see the large number of correlations that were run to examine the relationship between confidence/competence results and a range of demographic factors. As mentioned in my comment about the aims, this wasn't foreshadowed in the aims.

There were a considerable number of correlations run, but I did not see any corrections for multiple comparisons. The risk of Type I error was likely high. Several of the predictor variables (e.g., age, years of experience, education) were also likely highly correlated and there was no check for multicollinearity. I'd suggest that this is undertaken and further consideration be given to the variables included in the correlations.

Response: We appreciate the reviewer's careful attention. We have clarified the aims and analyses to ensure that the rationale for examining associations between confidence ratings and demographic/training factors is foreshadowed in the manuscript when discussing the aims in lines 131-139.
Analyses were re-run to adjust for Type I error as follows: First, Wilcoxon signed-rank tests comparing confidence levels across domains were run and adjusted with Bonferroni

correction. Next, we reduced dimensionality of the confidence ratings through exploratory factor analysis, yielding two composite scores which were then used as dependent variables in regression models. By modelling predictors simultaneously, we avoided inflating the number of pairwise correlations and reduced the risk of spurious findings. Moreover, multicollinearity among predictors was checked through Variance Inflation Factors (VIFs). The final regression models retained only education, years of experience, work setting, PD knowledge, referral practices, referral stage, and specialised training. The full description is reported in the analysis section in lines 205-225.

Others: As an aside, I was interested to read of the high proportion of SLPs that used AAC or a combination of behavioural and AAC approaches with their clients. This seemed a relatively high reported use of AAC compared with clinical practice in my own and nearby countries. I was interested to know more about what that entailed and why it was used.

Response: Thank you- This is indeed something that should be explored. Since we do not have further questions or open questions on the topic, we cannot do so with the current data, but we mention it in the limitations.

Reviewer: 2

1. **Abstract:**

-In the conclusion and elsewhere, the manuscript states "Malysian SLPs are competent in x". Consider using "feel competent", as this better reflects what is investigated. Also, responses to background questions suggest varying levels of knowledge: only 37% know at what disease stage speech changes can be detected, and 65% are familiar with micrografia.

Response: Thanks for pointing this out. We have revised to 'feel competent' in the current draft. As far as the background competence goes, we acknowledged some variability in lines 244-247. However, overall accuracy, which we chose to adopt as a measure of knowledge, was still high.

-In "Strengths and limitations" the manuscript mentions "use of a qualitative design, … provided rich insights". However, the study is quantitative. While qualitative methods could indeed have provided rich insights, the short (5-10 minutes to complete) survey with only two central questions offers more superficial–though still valuable–information.

Response: Thank you for this comment, we did remove this from the limitations section.

-In "Primary… outcomes" the stated goals do not align with the actual investigation. The outcomes mention identifying key themes and gathering recommendations, which suggests a qualitative approach. The study instead reports quantitative self ratings.

Response: We thank the reviewer for this important observation. Upon review, we agree that the original wording of the "Primary and Secondary Outcome Measures" section was misleading and did not accurately reflect our quantitative methodology. We have revised this section to align with the actual investigation conducted.

2. **Methods:**

-Motivation for the choice of survey questions is missing. It's great to adapt a survey from another study, but still some reasoning and references about content validity are needed. The inclusion of the seven questions on Parkinson's disease knowledge are not clearly explained–what does answering these correctly indicate about the respondents' knowledge?

Response: Thank you for your comments. The process of adapting the survey and the relevance of each section are now described in more depth in the "measures" section.

-Recruiting via a professional association and snowball sampling seems relevant. Were any reminders sent? Why not also via social media SLP groups? To assess how representative the results are, it would help to know approximately how many of the 380 SLPs in Malysia work with neurological patients.

Response: Thank you for these important methodological questions regarding our recruitment strategy. Yes, we sent follow-up reminders during the three-month data collection period. A reminder message was distributed via the Speech-Language Pathologists Association of Malaysia (SPEAK) email list and WhatsApp groups at the midpoint of data collection to encourage participation among eligible SLPs. The decision to recruit primarily through SPEAK's official channels (email and WhatsApp) rather than broader social media SLP groups was deliberate and based on several considerations:1) SPEAK represents the official professional body for Malaysian SLPs, ensuring we reached certified, practicing professionals who met our inclusion criteria (certified SLPs working in Malaysia with at least one active PD case).2) WhatsApp is widely used professionally by Malaysian SLPs for clinical communication and networking, making it an effective platform alongside email for reaching practicing clinicians. 3) Snowball sampling through professional networks allowed participants to share the survey through their own WhatsApp groups and contacts. This approach helped maintain data quality by ensuring participants were verified professionals rather than potentially unqualified respondents from open social media groups.

Regarding the representativeness of sample, the manuscript notes that approximately 380 SLPs work in Malaysia's government and private sectors (back in 2023). However, we acknowledge this is a limitation that should be explicitly addressed. Our survey required participants to have "at least one active case of PD in their caseload" as an inclusion criterion. While we can estimate our response rate at approximately 14% of all Malaysian SLPs (54/380), we do not have precise data on what proportion of the 380 SLPs actively work with neurological patients or specifically with PD patients.

We have added the recruitment process and clarification of the sample representativeness into the current draft for clarification.

We also added the following into the Discussion (Limitations): The study's representativeness is difficult to assess as national data on the number of Malaysian SLPs who work with PD patients or neurological populations is not available, limiting our ability to calculate a precise response rate among eligible clinicians.

3. Results:

-Titles of Figure 1, Table 2 and Table 3 are unclear and do not match the content. For example:

• Table 2 is described as showing "SLPs' perceptions of patient awareness and help-seeking behaviours", but it present "Current practices with PD"

• Table 3 is said to show "SLPs' perceived confidence in assessing patients with PD…" but it presents the SLPs' ratings on how competent they feel.

Response: Amended confidence to self-perceived competence throughout

-The description of the analysis should be placed at the end of the Methods section. Table 5 "Inferential statistics…" could be moved to the supplementary material. Main results should be summarized in the Results section, and statistical reporting results should follow the journal's format.

Response: Thanks for your comment. The methods section now includes an analyses section. As a consequence of running different analyses to address a comment, table 5 was removed, and all statistical results are discussed in the Results section, in text.

-Table 4 refers to "Confidence managing the risk involved in the rehab of PD". What are these risks?

Response: Thank you for the comment. We agree that, since it was not an open-ended question, and the participants could not respond to the specific types of risk, it is not particularly informative. For this reason, we removed it from the analyses.

-Figure and table titles are usually placed above the respective figure/table.

Response: Thank you, we amended accordingly to journal guidelines with titles above tables and below figures.

4. **Discussion:**

-The discussion on multilingualism is relevant, but more context about the situation in Malaysia would have been helpful, for example, number and distribution of languages, and how SLPs manage multilingualism in their practice, for example when it comes to language assessment and treatment.

Response: The point is well taken. Malaysia is a highly multilingual country with an estimated 130–140 living languages, including Malay as the national language, English as a widely used second language, and large Mandarin, Tamil and indigenous language communities distributed across Peninsular Malaysia and East Malaysia. In routine clinical practice, Malaysian SLPs typically prioritise assessment and treatment in the patient's functionally dominant language, draw on available Malay- and English-based tools, and supplement these with culturally and linguistically adapted informal or dynamic assessments when standardised tests are unavailable for a given language or dialect.

-SLP home services is mentioned, which may be beneficial for patients with Parkinson's disease. Maybe also mention studies about SLP tele practice?

Response: Agreed. We have added a comment on this in the discussion section to address it, lines 402-407.

5. **Manuscript**

-"Terminology: "Challenges" and "barriers" are used interchangeably, consider choosing one of the terms. The same applies to "competency" and "confidence".

Response: Thanks for your comment. We changed confidence to self-perceived competenece throughout, and challenges to barriers.

-Reference formatting is inconsistent. Only on page 6 is the "author, year" format used.
-Several in-text references are missing in the reference list.
-Reference list formatting varies.
Response: Amended

Reviewer: 3

This is an exploration of trends within the specific context of Malaysia. The analysis is fairly straightforward – describing the distribution of survey responses, and exploring correlations between variables. It seems to be that it is primarily a descriptive study –

there is no particular exposure-outcome association being tested, and the interpretation does not intend to generalize beyond this population.
The editor may have guidance on this, but I suggest possibly stating in the title or abstract that it is a descriptive analysis to make it clear how results should be interpreted.
The primary issue with the manuscript is that it does not adhere to the STROBE reporting guidelines, there are important elements of the methodology not reported. I have highlighted what I feel are the most important omissions below, but generally speaking, all of the comments and queries can be resolved by applying the STROBE checklist and following the guidance provided in the elaboration document: https://pubmed.ncbi.nlm.nih.gov/25046751/
Where relevant I have added a STROBE reference (e.g. “[item 1]”) to help the authors locate further information if needed.

Response: thank you. We have now used the CROSS guidelines recommended by the Editor (attached as a Supplemental File) and revised the manuscript accordingly.

Detailed comments:

1. **Abstract:**

-What is written is very clear. However, it isn’t immediately clear from the abstract alone whether the study is qualitative or quantitative. I suggest adding a section summarising the main statistical methods, and supporting the claimed results with statistical details.

Response: Thank you for the comment. We have now added an explicit description of the statistical methodology in section “Analyses”, and a mention to significance in the abstract.

2. **Introduction**

-Clearly written and informative, the introduction provides the context and rationale for this study. As a minor suggestion, the aims section is long, and clarity could be improved by numbering specific aims.

Response: We thank the reviewer for this helpful suggestion. We have revised the aims section to improve clarity by numbering specific aims, lines 132 and following.

3. **Methods:**

-I suggest applying more subheadings, relevant to each item in the STROBE reporting guidelines. This will help readers navigate and ensure that everything is reported in a logical order. As a suggested example, this could be “study design”, “setting”, “participants”, “variables”, “statistical analysis”.

Response: We have amended according to the STROBE guidelines as you suggested.

-It would help readers if the methods began with a high-level overview of the study design, setting, dates, and any other key information. Currently it begins with details of participants and procedures but this is harder to follow without an overview of the design. [item 4].
Response: Thanks for your comment. An overview of the study design, setting, and dates during which data were collected is now provided. Lines 152-158.

-Setting: I suggest adding more specifics of the setting so readers understand the context of the findings, e.g. where were participants working, what types of environment, how specifically was this setting targeted for recruitment? [item 5].

Response: Thanks for your comment. Since this was addressed by the participants themselves when answering the questions, it is discussed in the results section, lines 237 and following.

-Table 1. This table is helpful but is more suited to the results section.

Response: We have now moved information on participants of our study to the Results section.

-Variables: The second paragraph of the methods gives an overview of the survey from which variables were measured. But it would be helpful to know further how each of these relates to the aims, i.e. what was the purpose of measuring each category? For the key variables which the authors intend to analyse, it would be helpful to specify each item. Were any of these items combined or was each to be analysed separately? [item 7]

Response: Thanks for your comment. A detailed description of which variables were analysed statistically is now provided the “analysis” section.

-Statistical methods: This section does not meet STROBE reproducibility standards. Information should be provided so that readers can more or less predict what tables and figures they will see in the results, before viewing them. This aids readability and accurate interpretation by readers. I suggest adding a new paragraph or paragraphs, detailing (a) the software used (already reported), (b) that demographic characteristics were summarized (and how), (c) how practices of SLP with PD are summarized, (d) what comparisons or inferential statistics were generated – for which variables, how they relate to the aims, and the specific statistical tests.

Response: Thanks for your comment. The section called “analyses” now contains all of the relevant information.

4. **Results:**
-In each figure and table it is helpful to remind readers of the sample size, particularly when percentages are reported such as in figure 1.

Response: Amended.

-Table 3. I recommend adding the % sign to the percentages so these are not misinterpreted as anything else.

Response: Amended.

-Inferential statistics: As there was no time interval between measuring the demographic characteristics and reported competence levels, it is not possible to determine cause and effect. Also because no modelling is performed to adjust for external factors which could influence both variables. I suggest reframing this part of the analysis as exploring correlations between variables, and removing reference to "influences", "predicted" etc, as this language is not supported by the data type.

Response: Thanks for your comment. In response to this comment and one by another reviewer, different analyses were run, including correlations among competence variables. We kept an analysis that checked the weight of demographic factors as predictors (used in its statistical meaning) of potential differences, but we added comments in the limitation section regarding the limited generisability of the results given the potential external factors and limited variability.

-To align with STROBE reporting guidelines, please support the in-text results with statistics, for example: "Age and years of experience were the most significant factors, with older and more experienced participants feeling more competent in assessing all aspects and managing speech and language as well as more competent in managing risks" report the mean or median confidence levels for the different age groups to demonstrate this.

Response: Thanks for your comment. Analyses are now reported in text.

-Table 5. A footnote should explain what each statistic means, e.g. U, r, H(4). For some it is not really clear how a comparison was made or what the statistic represents, because the variables are not continuous – adding information in the methods would help clarify this.

Response: Thank you for the comment. Table 5 is no longer relevant and statistics are discussed in text.

1. **Discussion**

-There are currently no strengths or limitations stated. I suggest the major limitation and caveats for the interpretation which should be noted are that (a) much of it is a descriptive analysis, and as such is specific to this population and (b) the comparisons which were

made are all bivariate – no adjustment was made for external factors (confounding), and so the estimated correlations could be biased, and (c) only correlations can be determined, not causality, due to the lack of a temporal distinction between variables.

Response: We thank the reviewer for this important observation. We agree that the Discussion section should explicitly address the study's strengths and limitations with particular attention to methodological constraints. We have added a dedicated subsection addressing these points.

VERSION 2 - REVIEW

**Reviewer**	**3**
**Name**	**Dack, Kyle**
**Affiliation**	**University of Bristol, MRC Integrative Epidemiology Unit**
**Date**	**16-Feb-2026**
**COI**	

Thank you to the authors, significant additions and revisions to improve the clarity of the methodology, which is now more transparent. The analysis appears reasonable and aligns with the interpretation and overall I would support the manuscripts publication. However, I have a few minor suggestions concerning the clarity of certain lines, or elements readers may struggle with, which the authors may wish to consider:

“Numbering of aims - We have revised the aims section to improve clarity by numbering specific aims, lines 132 and following.”

-It appears the aims have been revised, but there are not numbers, and it is still all a single large sentence from line 131 to 136. Possibly the numbering is RQ1 and RQ2? The following are minor suggestions and not critical to the manuscript, but it may be clearer if presented as a list with distinct sentences, as reviewer 1 also suggested. RQ1 also seems to encompass both (a) describing self-perceived competence, and (b) quantifying the relationship with other variables – this involves different statistical techniques and the results are presented in separate paragraphs, so I suggest these may be separate aims or sub-aims?

Line 155: “in the first half of year 2023” I suggest replacing with the specific date range, e.g. March 2023 to May 2023, to avoid any possibility of ambiguity. The specific dates are provided later on, but I don’t think repetition of the dates would be a negative.

Line 196: I think this sentence may be reporting the primary outcomes, but it is not stated as such and is a little confusing in its current form. Suggest adding “the primary aims were”, if I have interpreted it correct.

Statistical analysis: The approach is much clearer and overall looks reasonable. However, I suggest adding a couple of details to help readers:

-State the rationale for the modelling approach, it sounds like it is exploratory and descriptive, in which case EFA is suitable, but it is helpful to confirm so readers do not assume other purposes (e.g. inference).

-Report the Bonferroni corrected p-value threshold.

-RQ1 is described but not RQ2. Readers may notice this, so I suggest adding a brief sentence that it was summarised using response frequencies (number and percentages).

VERSION 2 - AUTHOR RESPONSE

Reviewer: 3
Dr. Kyle Dack, University of Bristol
Comments to the Author:
Thank you to the authors, significant additions and revisions to improve the clarity of the methodology, which is now more transparent. The analysis appears reasonable and aligns with the interpretation and overall I would support the manuscripts publication. However, I have a few minor suggestions concerning the clarity of certain lines, or elements readers may struggle with, which the authors may wish to consider:

“Numbering of aims - We have revised the aims section to improve clarity by numbering specific aims, lines 132 and following.”
-It appears the aims have been revised, but there are not numbers, and it is still all a single large sentence from line 131 to 136. Possibly the numbering is RQ1 and RQ2? The following are minor suggestions and not critical to the manuscript, but it may be clearer if presented as a list with distinct sentences, as reviewer 1 also suggested. RQ1 also seems to encompass both (a) describing self-perceived competence, and (b) quantifying the relationship with other variables – this involves different statistical techniques and the results are presented in separate paragraphs, so I suggest these may be separate aims or sub-aims? G

Yes, the numbering in question was supposed to be represented by the RQs in the previous version. However, we have now divided all steps of the results section into separate distinct sentences, listed from 1 to 4 to explicitly represent all analyses discussed in the results section (lines 133-140). These points are also discussed separately in the analyses section (lines 213-)

Line 155: “in the first half of year 2023” I suggest replacing with the specific date range, e.g. March 2023 to May 2023, to avoid any possibility of ambiguity. The specific dates are provided later on, but I don’t think repetition of the dates would be a negative.

Thank you for this helpful suggestion. We have updated the text to replace ‘in the first half of year 2023’ with the specific data collection period, which was from May 2024 to December 2024. We agree that including the precise dates improves clarity and avoids ambiguity.

Line 196: I think this sentence may be reporting the primary outcomes, but it is not stated as such and is a little confusing in its current form. Suggest adding “the primary aims were”, if I have interpreted it correct.

We decided to remove this sentence as it felt repetitive and not in line with what was being described in the section.

Statistical analysis: The approach is much clearer and overall looks reasonable. However, I suggest adding a couple of details to help readers:
-State the rationale for the modelling approach, it sounds like it is exploratory and descriptive, in which case EFA is suitable, but it is helpful to confirm so readers do not assume other purposes (e.g. inference).
-Report the Bonferroni corrected p-value threshold.

Thank you for this helpful suggestion. We have clarified the rationale for the modelling approach in the Statistical Analysis section, explicitly stating that the analyses were primarily exploratory and descriptive in nature, consistent with the aims. In addition, we have now explicitly reported the Bonferroni-corrected significance threshold used for the Wilcoxon signed-rank tests (α = 0.005, based on 10 pairwise comparisons), lines 222-226

-RQ1 is described but not RQ2. Readers may notice this, so I suggest adding a brief sentence that it was summarised using response frequencies (number and percentages).

Lines 235-236

Reviewer: 3
If you have selected 'Yes' above, please provide details of any competing interests.: NA
